# Differences in faecal microbiome composition between adult patients with UCD and PKU and healthy control subjects

**DOI:** 10.1016/j.ymgmr.2021.100794

**Published:** 2021-09-08

**Authors:** C. Timmer, M. Davids, M. Nieuwdorp, J.H.M. Levels, J.G. Langendonk, M. Breederveld, N. Ahmadi Mozafari, M. Langeveld

**Affiliations:** a Department of Dietetics and Nutritional science and Department of Endocrinology and Metabolism, Amsterdam University Medical Centers, Amsterdam, the Netherlands; b Department of Vascular Medicine, Amsterdam University Medical Centers, Amsterdam, the Netherlands; c Department of Dietetics and Department of Internal Medicine, Center of Lysosomal and Metabolic Diseases, Erasmus University Medical Center, Erasmus MC, Rotterdam, the Netherlands

**Keywords:** Microbiome, Gut, Faecal, Urea cycle defect, Phenylketonuria, Hyperammonemia, 16S rRNA, taxonomic marker genes, common to all bacteria, ADI, Arginine Deimination. Bacteria derive energy from the deamination of arginine to citrulline and citrulline cleavage to ornithine plus carbamoyl phosphate. The latter is then converted into ATP and carbon dioxide, or used for pyrimidine biosynthesis. This route also generates two moles of ammonia (one from the arginine-citrulline conversion, the second from carbamoyl phosphate hydrolysis*)*, Alpha Diversity, the species diversity in a microbial sample. Used to represent the taxonomic diversities of individual samples, Ammonium scavengers, agents developed for the reduction of blood ammonia concentration used for the treatment of patients with urea cycle disorders. Sodiumbenzoate and phenylbutyrate are ammonium scavengers, ASLd, argininosuccinate lyase (ASL) deficiency, ASSd, argininosuccinate synthetase (ASS) deficiency, ASV, Amplified Sequence Variant. A specific nucleotide sequence representing a bacterial lineage, ARG1d, arginase 1 (ARG1) deficiency, BCAA, branched chain amino acids: isoleucine, leucine and valine, DEGs, differentially expressed genes, DESeq, an R package to analyse count data from high-throughput sequencing assays such as RNA-Seq and test for differential expression, EAA supplement, essential amino acids supplement containing L-histidine, L-isoleucine, L-leucine, l-lysine, L-methionine, *L*-phenylalanine, L-threonine, L-tryptofaan and L-valine with optional L-cystine and L-tyrosine added (depending on what product is used), FPD, Faiths Phylogenetic Diversity, alpha diversity metric accounting for genetic diversity, Genus, a taxonomic rank, Metagenome, microbiome collective genome, OTCd, ornithine transcarbamylase deficiency, PFAA, precursor free amino acid supplement, in this case phenylalanine free, PKU, Phenylketonuria, PCoA, Principal Coordinate Analysis. PCoA is aimed at graphically representing a resemblance matrix between p elements (individuals, variables, objects, among others). By using PCoA we can visualize individual and/or group differences. Individual differences can be used to show outliers, Proteolytic capacity, the capacity to break proteins down into smaller polypeptides or amino acids. In this study: enzymes involved in protein degradation, RT-qPCR, real-time quantitative polymerase chain reaction, Sodium BPA, sodium phenylbutyrate, UCD, urea cycle defect

## Abstract

Urea cycle disorders (UCDs) are a group of rare inherited metabolic diseases causing hyperammonemic encephalopathy. Despite intensive dietary and pharmacological therapy, outcome is poor in a subset of UCD patients. Reducing ammonia production by changing faecal microbiome in UCD is an attractive treatment approach. We compared faecal microbiome composition of 10 UCD patients, 10 healthy control subjects and 10 phenylketonuria (PKU) patients. PKU patients on a low protein diet were included to differentiate between the effect of a low protein diet and the UCD itself on microbial composition. Participants were asked to collect a faecal sample and to fill out a 24 h dietary journal. DNA was extracted from faecal material, taxonomy was assigned and microbiome data was analyzed, with a focus on microbiota involved in ammonia metabolism.

In this study we show an altered faecal microbiome in UCD patients, different from both PKU and healthy controls. UCD patients on dietary and pharmacological treatment had a less diverse faecal microbiome, and the faecal microbiome of PKU patients on a protein restricted diet with amino acid supplementation showed reduced richness compared to healthy adults without a specific diet. The differences in the microbiome composition of UCD patients compared to healthy controls were in part related to lactulose use. Other genomic process encodings involved in ammonia metabolism, did not seem to differ. Since manipulation of the microbiome is possible, this could be a potential treatment modality. We propose as a first next step, to study the impact of these faecal microbiome alterations on metabolic stability.

**Take home message:**

The faecal microbiome of UCD patients was less diverse compared to PKU patients and even more compared to healthy controls.

## 1 Introduction

Urea cycle disorders (UCDs) are a group of rare inherited metabolic diseases causing hyperammonemic encephalopathy. Treatment of patients with UCDs consists of reducing nitrogen load (protein-restricted diet), improving residual urea cycle function (arginine and/or citrulline supplementation), removal of nitrogen by using alternative pathways (with drugs like sodiumbenzoate and/or phenylbutyrate) [1] and/or decreasing the intestinal production of ammonia and/or its absorption into the body (administration of antibiotics or lactulose) [2]. If natural protein tolerance is lower than the FAO/WHO/UNU 2007 safe levels of protein intake (0.83 g natural protein per kilogram bodyweight for adults), supplementation of essential amino acids (EAA) is given to ensure adequate amino acid availability for growth and maintenance [1].

Despite intensive dietary and pharmacological therapy, outcome is poor in subset of UCD patients [1], [3], with frequent hyperammonemic decompensations and impaired physical functioning and poor quality of life. In these patients liver transplantation could be considered as a last resort [4], [5].

Under normal conditions, gut bacteria produce a significant part of ammonia circulating in the body [6], with predictions varying between 20 and > 50% [7]. In healthy individuals, the composition of the faecal microbiome can be significantly influenced by diet [8], [9]. In the first years of life, the diversity of the faecal microbiome increases. During adult life, the core composition remains relatively constant [8]. UCD patients are treated with a protein restricted diet that greatly differs from a healthy diet of ‘normal’ diet. To ensure sufficient caloric intake, UCD patients eat more carbohydrates, and essential amino acids are supplemented. All these elements separately can change the faecal microbiome composition [10], [11], [12], [13], [14], [15], [16]. Higher carbohydrate intake can reduce faecal microbiome diversity [17]. In vitro growth of human intestinal bacteria on a mixture of amino acids results in enrichment of pathogenic species such as *Escherichia Coli* and *Shigella* [12]. These bacteria are known to produce ammonia [13]. In the colon, ammonia can be generated by microbial fermentation of glutamine, serine, threonine, and glycine [14]. In addition, the use of l-carnitine, as well as lactulose (regularly used by UCD patients and not by PKU patients) can have an effect on faecal microbiome composition. Overall, changes in microbiome composition in UCD patients can be expected, and compositional and functional shifts of the microbiome towards ammonia production can potentially have a negative effect on metabolic control.

From a treatment perspective, reducing ammonia production by faecal microbiome as a way of improving metabolic control in UCD, is an attractive approach [2]. In a mouse model of hepatic injury, introduction of an engineered microbiota with reduced urease activity decreased gut ammonia production, improved morbidity and reduced mortality [18]. Administration of probiotics may have a positive effect on clinical manifestations in patients with hepatic encephalopathy, though well designed trials of sufficient size are lacking [19]. In UCD patients, currently a phase II trial is conducted studying the effect of a chemical compound (KB195, Kaleido Biosciences Inc., Lexington, Massachusetts, ClinicalTrials.gov Identifier: NCT03933410) that aims to change faecal microbiome composition to reduce ammonia production (preliminary safety and clinical data presented at the SSIEM 2019 conference) [20]. In addition, supplementation of *A. muciniphila* seems safe in humans [21], this might also be a good candidate to test in UCD.

There is no information available on faecal microbiome composition in UCD patients. The overall microbiome of an OTC deficiency (the most prevalent UCD) mouse model (the spf-ash mouse) differed significantly from that of the wild type mice [22]. In another inborn error of protein metabolism, phenylketonuria (PKU), human studies on microbiome composition have been performed. In PKU, accumulation of a single amino acid, phenylalanine, results in neurotoxicity, without hyperammonemic decompensation. PKU is also successful treated with a natural protein restricted diet, supplemented with phenylalanine free amino acid (PFAA) mixtures. Faecal microbiome analysis in children with PKU showed that the PKU microbiome differed significantly from healthy children on a normal diet [16], [23].

In summary, the faecal microbiome of UCD patients is expected to differ significantly from healthy control subjects due to dietary and medical treatment. If these differences affect the gut ammonia production, this could have an effect on metabolic stability of these patients.

We studied and compared faecal microbiome composition of UCD patients, PKU patients and healthy control subjects (CON). PKU patients were included to differentiate between the effect of a low protein diet and the UCD itself on microbial composition. In addition to the overall microbiome composition we focussed on metagenomic attributes of ammonia metabolism and looked at differences in microbiome composition between stable conditions and hyperammonemic decompensation in a subset of patients. We also analyzed the amount of short chain fatty acids (SCFAs) in the faecal samples, as we know that SCFAs are produced in the colon by bacterial fermentation of dietary fibers and resistant starch. SCFAs are essential molecules, involved not only in host metabolism and immunity, but also in intestinal barrier function [24].

Hypothesis: In urea cycle defect patients, the protein restricted diet in combination with essential amino acid supplementation, results in a different faecal microbiome composition compared with healthy individuals without a specific diet.

### 1.1 Aims

- To detect differences between faecal microbiome composition of UCD patients, PKU patients and healthy controls.
- To study the relative abundance of ammonia producing bacterial species in the microbiome of UCD patients versus healthy controls and UCD patients versus PKU patients.
- To study the influence of UCD itself versus the protein restriction and amino acid supplementation on microbiome composition by comparing UCD and PKU patient outcomes.

## 2 Methods

Since no previous studies on microbiome composition in UCD patients have been carried out, power calculation was based on a study looking at differences in the abundance of bacterial species in patients with liver cirrhosis with encephalopathy versus healthy control subjects [18]. To pick up the reported 7.3% difference in relative abundance of Firmicutes Clostridiales (known to produce ammonia) we need a sample size of at least 6 subjects per group (2-sided chi-square test with a desired alpha of 0.05 and a desired power of 0.8). To be able to pick up differences in other bacterial strains as well, we included as many patients possible (estimated were 15 eligible UCD patients at the Erasmus and Amsterdam Medical Centers combined). Between 2017 and 2019 adult UCD and PKU patients, attending the outpatient clinic for inherited metabolic diseases of Erasmus MC and Amsterdam UMC, location AMC, were asked to participate. Inclusion criteria were a definite diagnosis of classic PKU or UCD, treatment with a low protein diet (less than 0.83 g protein/kg/day) and use of amino-acid supplementation. Exclusion criterion was antibiotics use in the 3 months preceding inclusion. From 3 UCD patients a second stool sample was collected at hospital admission for hyperammonemic encephalopathy. Healthy adults were recruited via advertisements in 2018–2019. These healthy controls were unrelated to the patients, and not sharing a household. The inclusion criteria were: age over 18 years and the ability to give informed consent. Exclusion criteria were a specified diet and antibiotics, probiotics or laxatives intake or other medication likely to influence gut transit time in the 3 months preceding inclusion. All participants, or their legal representative, gave written informed consent. The study was approved by the regional ethics committees (Amsterdam UMC, location AMC and Erasmus MC) and followed the Helsinki Declaration.

Questionnaires were used to provide information on height, weight and a detailed 1 day dietary record. The first faecal sample on the next day was collected by participants. Faecal samples were cooled by a frozen cooling element and send to AMC until essay. All samples were stored at −80 °C within 24 h.

### 2.1 Microbiome sample and data processing

DNA was extracted from faecal material using a repeated bead beating protocol. DNA was purified using Maxwell RSC Whole Blood DNA Kit. 16S rRNA gene amplicons were generated using a single step PCR protocol targeting the V3-V4 region. PCR products were purified using Ampure XP beads and purified products were equimolar pooled. The libraries were sequenced using a MiSeq platform using V3 chemistry with 2 × 251 cycles.

Forward and reverse reads were truncated to 240 and 210 bases respectively and merged using USEARCH. Merged reads that did not pass the Illumina chastity filter, had an expected error rate higher than 2, or were shorter than 380 bases were filtered. Amplified Sequence Variants (ASVs) were inferred for each sample individually with UNOISE3 with a minimum abundance of 4 reads. Unfiltered reads were than mapped against the collective ASV set to determine the abundances. Taxonomy was assigned using the RDP classifier and SILVA 16S ribosomal database V132. Contaminants were identified using decontam software and subsequently, together with lab specific known contaminants, removed from the dataset. The parameters we obtained were: relative abundance and alpha diversity (observed diversity, Shannon index and Phylogenetic diversity) as further described in supplementary file 1.

Three different sets of enzymes may serve as proxy read out for microbial ammonia production, namely those involved in protein degradation (EC:3.4.-.-), ammonia lyase (EC:4.3.1.-) and amino acid oxidoreductase (EC:1.4.-.-). Phylogenetic Investigation of Communities by Reconstructing Unobservable States (Picrust2) was used to infer metagenomic attributes involved in ammonia metabolism: proteases, ammonia-lyases, amino acid oxidoreductase, urease, carbamoyl-phosphate synthase and glutamine synthase.

Sample preparation and HPLC analysis of the faecal SCFA's samples were carried out according to the method from De Beare et al. [25] with some modifications as described in supplementary file 2.

### 2.2 Data analysis

Faecal microbiome data was analyzed and visualized in R (V3.6.3). Phyloseq and picante were used to calculate alpha diversity metrics which were tested using ANOVA. Differences in enzyme and pathway abundance were also tested using ANOVA. Adonis implemented in Vegan was used to test differences in composition for various patient characteristics. Differential abundance of taxa was tested using DESeq2. Obtained *p*-values were FDR (false discovery rate) corrected.

The macronutrients and energy intake was calculated using the ‘*eetmeter*’ [26]: a food calculator tool developed by an independent organisation: the Netherlands Nutrition Centre. Information on prescribed medication, amino acid supplement use and dietary advice came from the electronic patient records. For underweight (BMI < 18.5) and overweight (BMI > 27.5) participants, the adjusted bodyweight was also calculated. Differences between groups (UCD, PKU and CON) were tested using Kruskal-Wallis tests. Data are presented as medians and range. These analyses were performed using SPSS version 26.0 software (IBM Corp., Armonk, NY, USA); a *P*-value <0.05 was considered statistically significant, Bonferroni adjusted for multiple testing.

## 3 Results

A total of 30 participants were enrolled in the study: 10 UCD patients, 10 PKU patients and 10 healthy volunteers. The data of one UCD patient was excluded from the microbiome analyses, because after inclusion this patient used antibiotics before faecal sampling. From three UCD patients an additional second faecal sample was collected during hyperammonemic decompensation.

### 3.1 Patient characteristics

Of the ten UCD patients, three were Ornithine transcarbamylase (OTC) deficient patients, four were Argininosuccinic acid lyase (ASL) deficient patients, two were Argininosuccinic acid synthetase (ASS1) deficient patients and one had Arginase deficiency (ARG1). Patient characteristics are shown in Table 1. There were no significant differences between the three groups in age, sex, length, weight, adjusted body weight, BMI, energy intake, fat intake and the use of citrulline, phenylbutyrate and macrogol. The natural protein intake was lower and the intake of amino acid supplements was higher in PKU patients compared to UCD patients (*p* = 0.003), as can be expected when following the management guidelines for these disorders [1], [27]. The carbohydrate intake was highest in UCD patients. As part of the disease management, the use of arginine, sodiumbenzoate and levocarnitine was highest in UCD patients. UCD patients also used more laxatives compared to healthy controls. Individual patient characteristics are shown in supplementary Table 2. Differences in diets between the 3 groups are displayed in supplementary Table 3.

### 3.2 Faecal microbiome composition

**Fig. 1:**
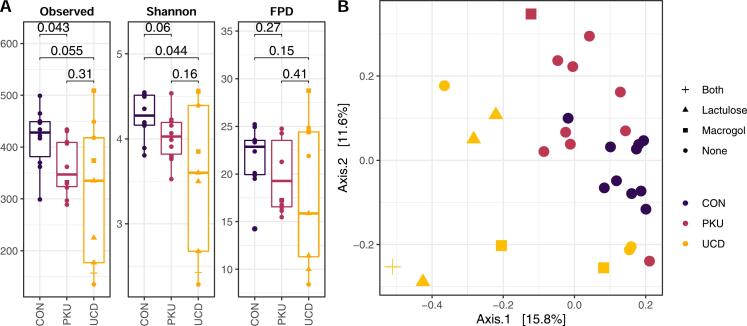
A. Alpha diversity measurements of microbial communities in the Urea cycle defect, phenylketonuria and control groups. Each panel represents one alpha diversity measure as follow: Observed = total number of operational taxonomic units observed; Shannon = microbial indexes of diversity. FPD: Faith's phylogenetic diversity. Boxes span the first to third quartiles; the horizontal line inside the boxes represents the median. Whiskers extending vertically from the boxes indicate variability outside the upper and lower quartiles, and the single coloured dots indicate outliers. CON = healthy controls, PKU = patients with phenylketonuria, UCD = patients with a urea cycle defect. Fig. 1B: Principal coordinate analysis (PCoA) of the Bray-Curtis dissimilarity of the microbiome of UCD and PKU patients and healthy control subjects. Samples were plotted on the first two principal coordinates, with colours for health condition, and shape for type of laxative. CON = healthy controls, PKU = patients with phenylketonuria, UCD = patients with a urea cycle defect.

There was lower richness of the faecal microbiome of PKU patients compared to healthy controls (*p* = 0.043). Shannon diversity was lower in faecal microbiome of UCD patients compared to control subjects (*p* = 0.044). There were no statistically significant differences in faecal microbiome richness or diversity between the PKU and UCD groups. Phylogenetic diversity did not differ between the three groups (Fig. 1A). Principal coordinate analysis of the microbiome Bray-Curtis dissimilarity shows almost complete separation of the UCD group and the other 2 groups, and grouping accounts for 15% of the observed variance between the 3 groups (Fig. 1B; permanova; *p* = 0.001; R2 = 0.15).

**Table 1:** Patient group characteristics and differences between UCD patients, PKU patients and healthy controls.

	UCD	PKU	CON	Difference
N	9	10	10	NS
Age (year)	32 (19–65)	35.5 (19–50)	35.5 (20–58)	NS
Length (cm)	170 (150–199)	170 (156–187)	179 (165–194)	NS
Bodyweight (kg)	66.6 (39.5–85.0)	70.7 (50.0–90.1)	77.9 (56.0–90.0)	NS
Adjusted bodyweight (ABW) *	66.1 (44.4–84.7)	70.7 (53.5–79.5)	77.9 (56.0–90.0)	NS
BMI (kg/m^2^)	21.7 (15.8–32.2)	24.3 (17.3–31.4)	23.9 (19.8–26.0)	NS
Protein advice (g/day)**	36 (22–53) ^#^	15.5 (9–23) ^#^	n.a.	Sign: PKU vs UCD (0.000)
Protein intake (g/day)***	31 (20–63) ^$^	19 (11–39) ^±^	83 (30–119) ^$ ±^	Sign: UCD vs CON (0.038); PKU vs CON (0.000)
Protein intake/ kg ABW (g/kg/day)	0.5 (0.3–1.3)	0.3 (0.2–0.6) ^±^	1.1 (0.4–2.1) ^±^	Sign: PKU vs CON (0.000)
Protein from AA suppl./day)	11 (0−20) ^#^	60 (45–80) ^± #^	0 (0–0) ^±^	Sign: UCD vs PKU (0.015); PKU vs CON (0.000).
Energy intake (kCal/day)	1987 (1212–3760)	1820 (1417–2845)	1985 (1263–2586)	NS
Carbohydrate intake (g/day)	328 (192–710) ^$^	219 (153–436)	198 (85–326) ^$^	Sign: UCD vs CON (0.037)
Fat intake (g/day)	48 (36–102)	54 (24–120)	79.5 (25–112)	NS
Arginine supplement (g/day)	6 (0–6) ^$ #^	0 (0–4) ^#^	0 (0–0) ^$^	Sign: UCD vs CON (0.008); UCD vs PKU (0.030)
Citrulline supplement (g/day)	0 (0–9)	0 (0–0)	0 (0–0)	NS
Sodiumbenzoate (g/day)	12 (0–16) ^$ #^	0 (0–0) ^#^	0 (0–0) ^$^	Sign: UCD vs CON (0.000); UCD vs PKU (0.000)
Phenylbutyrate (g/day)	0 (0–15)	0 (0–0)	0 (0–0)	NS
Lactulose (ml/day)	0 (0–90) ^$ #^	0 (0–0) ^#^	0 (0–0) ^$^	Sign: UCD vs CON (0.018); UCD vs PKU (0.018)
Levo-carnitine (mg/ day)	1000 (0−3000) ^$ #^	0 (0–0) ^#^	0 (0–0) ^$^	Sign: UCD vs CON (0.000); UCD vs PKU (0.000)

All outcomes are presented as median with their range. Kruskal-Wallis test with Bonferroni correction was performed. Statistical differences (2-sided tested) are shown as: differences # between UCD and PKU; $ between UCD and CON; ± between PKU and CON. A *p* < 0.05 level is considered significant.

UCD = urea cycle defect patients. PKU = phenylketonuria patients. CON = healthy controls. BW = bodyweight. BMI = body mass index. Sign = significant. NS = not significant. *ABW = Adjusted BW (weight adjusted to BMI 18,5–27,5 when beneath or above). ** Advised natural protein intake (g/day). *** reported natural protein intake (g/day). **** protein equivalent from AA supplementation (g/ day).

Most UCD patients (*n* = 6; 67%) used laxatives (33% Lactulose, 22% Macrogol, and 11% both), and one of the 10 PKU patients (10%) in our cohort used Macrogol. Laxative use was the main driver of the observed differences in microbiome composition between UCD patients, PKU patients and healthy controls (*p* = 0.003; R2 = 0.10). Other parameters that had an association with faecal microbiome composition were the use of arginine and carnitine supplementation (*p* = 0.011; R2 = 0.074, *p* = 0.023; R2 = 0.066) (Supplementary Fig. 4). After correcting for laxative use, microbiome composition was still statistically significant different between UCD patients versus both PKU patients and controls (*p* = 0.015; R2 = 0.1).

**Fig. 2:**
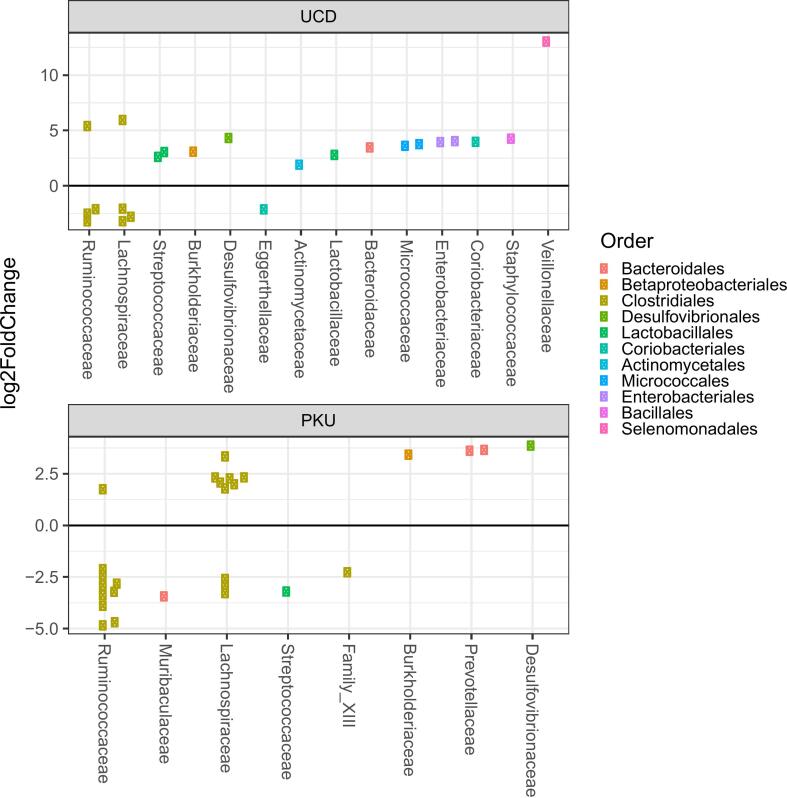
Changes in microbial abundance by calculating log2-fold changes in the relative abundance compared to healthy controls.

Several taxa showed significant differential abundance between healthy control subjects and UCD patients and between healthy control subjects and PKU patients (Fig. 2).

Compared to control subjects, the microbiome of UCD patients shows a reduced abundance of strictly anaerobic Clostridia and increased abundance of facultative anaerobic clades, characteristic for the microbiome of the upper GI tract. Differences between PKU and control subjects is characterized by changes in the order of *Clostridiales*. The *Lachnospiraceae* are more prevalent in the microbiome of PKU subjects compared to the microbiome of healthy control subjects, the *Ruminococcaceae* are less prevalent compared to the microbiome of healthy control subjects.

### 3.3 Microbiome ammonia metabolism

**Fig. 3:**
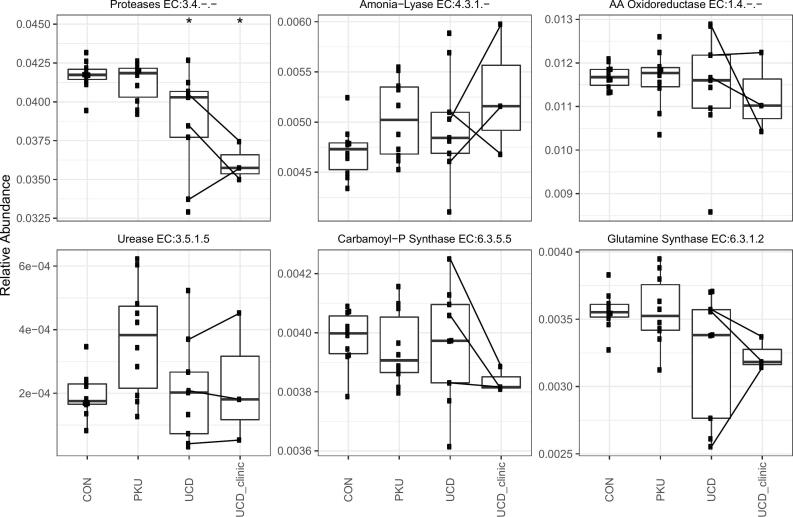
Boxplots of the functional abundance of the enzyme activity of faecal microbiome for processes involved in ammonia metabolism. Comparing healthy controls (CON), phenylketonuria patients (PKU), urea cycle defect patients (UCD) and UCD patients during a hyperammonemic decompensation (UCD_clinic). The black lines connect the measurements of the same patient in stable and in decompensated state. * indicates significant difference (*p* < 0.05) compared to healthy controls.

The functional characteristics of the microbiota involved in ammonia metabolism are displayed in Fig. 3.

Overall the results showed a decrease in proteolytic capacity (enzymes involved in protein degradation) in UCD patients compared to healthy controls, that may be even more pronounced during hyperammonemic decompensation. Ammonia-lyase and amino acid oxidoreductase potential do not differ. Urease potential (EC:3.5.1.5) also did not differ between the three groups. Glutamine synthase (GS: EC:6.3.1.2) and carbamoyl-phosphate synthase (CPS: EC: 6.3.5.5) showed no differences between the three groups.

The total protein intake (natural protein plus disease specific L-amino acid based protein substitutes) of UCD patients was on average 51% of the protein intake of healthy controls, and 53% of the total protein intake of PKU patients. The total protein intake of PKU patients (natural protein and PFAA) was 95% of healthy controls and therefor in the normal range.

### 3.4 Faecal short chain fatty acids (SCFA)

There was no statistically significant difference in faecal SCFA composition between the three subgroups (UCD, PKU, CON).

## 4 Discussion

The microbiome of UCD patients is significantly different when compared to PKU patients and healthy control subjects. The obligate treatment for both UCD and PKU patients is a low natural protein diet, complemented with amino acid supplementation for sufficient protein intake. Laxatives are frequently used in the UCD group and are well-known to change microbiome composition [2], [13]. The major difference in microbiome content between the studied groups is associated with lactulose use, which was only used by UCD patients. The faecal microbiome of those UCD patients contain an increased abundance of facultative anaerobic clades, known to be associated with a short intestinal transit time. As the use of arginine and carnitine supplementation was associated with both differences in faecal microbiome as well as laxative use, the observed difference in microbiome composition may be due to collinearity. As reported previously [16], [23], [28], the faecal microbiome of PKU patients differs from that of healthy control subjects. In this study we show for the first time a different faecal microbiome in UCD patients, compared to both PKU and healthy controls. Healthy control subjects have the most rich and diverse faecal microbiome of the 3 groups, while PKU patients showed a reduced richness and UCD patients a less diverse microbiome. A less rich and/or diverse microbiome is associated with different health issues, such as low-grade inflammation, obesity and metabolic syndrome [17], [29], [30]. Whether the altered faecal microbiome diversity affects the health or whether it is a disease marker is still unknown. The impact of these presented findings on morbidity and complications in UCD and PKU patients cannot be given based on the presented findings, this requires a prospective and larger study.

In healthy adults, bacteria belonging to the families *Lachnospiraceae* and *Ruminococcaceae* co-dominate the faecal bacteria [31]. *Ruminococcaceae* are known butyrate producers, while *Lachnospiraceae* are mainly propionate producers. Butyrate and propionate are two of the main short chain fatty acids (SCFA) metabolites. We found a higher prevalence of several members of *Lachnospiraceae* in the faecal microbiome of PKU patients, whilst multiple lineages of *Ruminococcaceae* species were reduced. The increased abundance of *Lachnospiracea* was also seen in the faecal microbiome of pigs fed with a comparable diet to PKU patients: a low natural protein diet supplemented with amino acids [32]. Two studies with PKU patients reported opposite findings: both reported reduced prevalence of *Lachnospiraceae* [16], [28]. In the study of Pinheiro de Oliveira [16] some patients used antibiotics (25% of the PKU group and 30% of the CON group used antibiotics in the 6 months preceding the faecal sample collection) and all patients were children. Antibiotics might explain the contrasting findings. Also the microbiome of children is different from adults [33]. The study by Mancilla [28] doesn't report antibiotic or other medication use. Both our study and these two studies were performed in small groups, with 8–10 patients per group, which can result in type 2 errors. The large intra individual variations in specific groups of bacteria can also result in type 2 errors.

The inferred genomic capacity of the faecal microbiome composition shows a selection for a less proteolytic microbiota in UCD patients. This was not observed in PKU patients, suggesting that the total protein intake (natural protein plus PFAA) may be used as a protein source by the proteolytic members of the faecal microbiome.

In case of reduced protein intake, the microbiota might need to resort to amino acid biosynthesis in order to meet their amino acid requirements. Glutamine synthase and carbamoyl-phosphate synthase are critical for activation and fixation of ammonia and amino acids biosynthesis. Our results showed no differences in the capacity between the three groups based on genetic analysis. However, since these functions are highly conserved throughout the bacterial kingdom, they are likely to be regulated at protein expression levels.

The other processes involved in ammonia metabolism that we tested did not differ in the presented study groups. However, fluxes through these pathways can also be regulated at the protein level, in addition to genetic prevalence. So the net effect of the gut ammonia production cannot be estimated in the current study.

Adding peptides and amino acids to faecal bacteria from healthy humans in vitro resulted in increased richness of various (pathogenic) bacterial species [12], [34].

The amino acid supplementation of UCD patients may contain separate citrulline or arginine doses. Gut bacteria can produce energy with arginine deaminase, whilst generating ammonia as a waste, comparable to well-known urease producing bacteria [35]. In the presented analysis the genes encoding for enzymes involved in ammonia metabolism (such as arginine deaminase) were not increased in the faecal microbiome of UCD patients, compared to PKU patients and healthy controls.

As Levitt et al. discuss, the major source of plasma ammonia is the GI tract [7]. Another contributing factor besides urea degradation is the use of systemic glutamine. We hypothesize that a significant amount of glutamine may be used to produce ammonia rich mucus glycoprotein. These glycoproteins are secreted into the gut, where they serve as a protective layer. Glycoproteins are metabolized by gut microbes and contribute to gut ammonia production. Changes in mucus microbiome homeostasis may thus contribute to metabolic stability in UCD patients. This needs further research.

There are several limitations of the current study. A single 1-day food record does not account for day-to-day variation. Another limitation is that dietary fibre content was not assessed despite the known effect of fibers on microbiome. The study groups are relatively small and we only studied faecal microbiome composition, without looking at mucus layer composition. Total bacterial load and altered microbial activity might have large effects on gut ammonia metabolism. The aim was to demonstrated differences in microbiome in patients on a low natural protein diet, but the outcome in the two groups differed and a significant confounding effect of laxative use was demonstrated (Supplementary Fig. 4). In future studies, this bias could be partly eliminated by prescribing lactulose to the control group as well as the PKU patient group before stool collection. It would also be interesting to know the changes in faecal microbiome composition in UCD patients precede hyperammonemic decompensation, to identify the causal relationship between faecal microbiome and hyperammonemic decompensation. In this study, we did not investigate the differences in mucus composition of UCD versus PKU patients or healthy controls. As mucus might substantially contribute to the gut nitrogen pool, and thus to the ammonia metabolism, this needs further investigation in the future. The presented results were obtained in a cross-sectional design, and only 3 samples were obtained during hyperammonemic decompensation. In future research, longitudinal data collected in a relative stable phase as well as before and during hyperammonemic decompensation, might give further insight in the interaction between the microbiome and hyperammonemic decompensation. Determining ammonia-lyases and amino acid oxidoreductase activity will be a better proxy for amino-acid fermentation then the PICRUSt2 inferred genomic blue print as a potential measurement.

## 5 Conclusion

Adult UCD patients and PKU patients, both on a low protein diet with amino acid supplementation, have a different faecal microbiome composition compared to healthy controls without a specific diet. Healthy control subjects have the most rich and diverse microbiome of the 3 groups. The differences in the faecal microbiome composition of UCD patients compared to healthy controls are in large part explained by lactulose use. Whether the microbiome alterations influence metabolic stability in UCD patients, and whether manipulation of the microbiome is a potential treatment modality, should be determined in future studies.

The following are the supplementary data related to this article.Supplementary Fig. 4 Visual correlation matrix: a graphical display of correlation, with confidence interval. Positive correlations are displayed in red and negative correlations in blue colour. Colour intensity and the size of the circle are proportional to the correlation coefficients. The most right column shows the explained variance (R2) in microbiome composition as determined by permutation MANOVA. Supplementary Fig. 4

Supplementary Table 2 Individual patient characteristics of UCD patients, PKU patients and healthy controls. Supplementary Table 2

Supplementary Table 3 Differences in the major dietary items, laxative use and microbiome specifics between UCD patients, PKU patients and healthy controls. Supplementary Table 3

Supplementary file 1 A layman explanation of microbiome terminology and data-analyzation. Supplementary file 1

Supplementary file 2 HPLC Short Chain Fatty Acid Analysis. Supplementary file 2

## Funding

This study was supported by ESN, the Dutch Society for Inborn Errors of Metabolism. M.N. is supported by a personal ZONMW VICI grant 2020 [09150182010020].

## Data availability

Raw sequence reads were submitted to the European Nucleotide Archive (ENA) and can be found under study PRJEB41032.

## Author contributions

CT and ML participated in the planning and conducting of the project. CT, JGL, NAM and MB participated in acquisition of data. MD analyzed and reported the microbiome data and provided the figures. JHML analyzed and reported the SCFA data. CT wrote the first draft of the manuscript and provided the final approval for the submission. All authors have participated in drafting the manuscript or revising it critically for important intellectual content. All authors provided approval for the submission.

## Declaration of Competing Interest

CT, MD, JHML, JGL, MB and NAM declare to have no conflict of interest. ML is involved in premarketing studies with Genzyme, Protalix and Idorsia. MN is in the Scientific Advisory Board of Caelus Pharmaceuticals, the Netherlands and Kaleido, USA. None of these are directly relevant to the current paper.

## References

[1] Haberle J., Burlina A., Chakrapani A. (2019). Suggested guidelines for the diagnosis and management of urea cycle disorders: first revision. J. Inherit. Metab. Dis..

[2] Liu J., Lkhagva E., Chung H.J., Kim H.J., Hong S.T. (2018). The pharmabiotic approach to treat hyperammonemia. Nutrients.

[3] Posset R., Gropman A.L., Nagamani S.C.S. (2019). Impact of diagnosis and therapy on cognitive function in urea cycle disorders. Ann. Neurol..

[4] Kolker S., Valayannopoulos V., Burlina A.B. (2015). The phenotypic spectrum of organic acidurias and urea cycle disorders. part 2: the evolving clinical phenotype. J. Inherit. Metab. Dis..

[5] Walker V. (2009). Ammonia toxicity and its prevention in inherited defects of the urea cycle. Diabetes. Obes. Metab..

[6] Williams R. (2007). Review article: bacterial flora and pathogenesis in hepatic encephalopathy. Aliment. Pharmacol. Ther..

[7] Levitt D.G., Levitt M.D. (2018). A model of blood-ammonia homeostasis based on a quantitative analysis of nitrogen metabolism in the multiple organs involved in the production, catabolism, and excretion of ammonia in humans. Clin. Exp. Gastroenterol..

[8] Wu G.D., Chen J., Hoffmann C. (2011). Linking long-term dietary patterns with gut microbial enterotypes. Science.

[9] David L.A., Maurice C.F., Carmody R.N. (2014). Diet rapidly and reproducibly alters the human gut microbiome. Nature.

[10] Fan W., Tang Y., Qu Y., Cao F., Huo G. (2014). Infant formula supplemented with low protein and high carbohydrate alters the intestinal microbiota in neonatal SD rats. BMC Microbiol..

[11] Shortt C., Hasselwander O., Meynier A. (2018). Systematic review of the effects of the intestinal microbiota on selected nutrients and non-nutrients. Eur. J. Nutr..

[12] Richardson A.J., McKain N., Wallace R.J. (2013). Ammonia production by human faecal bacteria, and the enumeration, isolation and characterization of bacteria capable of growth on peptides and amino acids. BMC Microbiol..

[13] Vince A.J., Burridge S.M. (1980). Ammonia production by intestinal bacteria: the effects of lactose, lactulose and glucose. J. Med. Microbiol..

[14] Ramezani A., Massy Z.A., Meijers B., Evenepoel P., Vanholder R., Raj D.S. (2016). Role of the gut microbiome in uremia: a potential therapeutic target. Am. J. Kidney Dis..

[15] Al-Zyoud W., Nasereddin A., Aljarajrah H., Saket M. (2019). Culturable gut bacteria lack Escherichia coli in children with phenylketonuria. New Microbes New Infect.

[16] Pinheiro de Oliveira F., Mendes R.H., Dobbler P.T. (2016). Phenylketonuria and gut microbiota: a controlled study based on next-generation sequencing. PLoS One.

[17] Hills R.D., Pontefract B.A., Mishcon H.R., Black C.A., Sutton S.C., Theberge C.R. (2019). Gut microbiome: profound implications for diet and disease. Nutrients.

[18] Shen T.C., Albenberg L., Bittinger K. (2015). Engineering the gut microbiota to treat hyperammonemia. J. Clin. Invest..

[19] Rivera-Flores R., Moran-Villota S., Cervantes-Barragan L., Lopez-Macias C., Uribe M. (2020). Manipulation of microbiota with probiotics as an alternative for treatment of hepatic encephalopathy. Nutrition.

[20] Haberle J.T.A., Sawicki E., Mahowald M., Meehan B., Beccarelli A., Weber K., Koziel M.J. (2019). An open-label, single-arm clinical study to evaluate safety and tolerability of KB195, a novel glycan in patients with urea cycle disorders. J. Inherit. Metab. Dis..

[21] Depommier C., Everard A., Druart C. (2019). Supplementation with akkermansia muciniphila in overweight and obese human volunteers: a proof-of-concept exploratory study. Nat. Med..

[22] Allegri G., Deplazes S., Rimann N. (2019). Comprehensive characterization of ureagenesis in the spf(ash) mouse, a model of human ornithine transcarbamylase deficiency, reveals age-dependency of ammonia detoxification. J. Inherit. Metab. Dis..

[23] Bassanini G., Ceccarani C., Borgo F. (2019). Phenylketonuria diet promotes shifts in firmicutes populations. Front. Cell. Infect. Microbiol..

[24] Farre R., Fiorani M., Abdu Rahiman S., Matteoli G. (2020). Intestinal permeability, inflammation and the role of nutrients. Nutrients.

[25] De Baere S., Eeckhaut V., Steppe M. (2013). Development of a HPLC-UV method for the quantitative determination of four short-chain fatty acids and lactic acid produced by intestinal bacteria during in vitro fermentation. J. Pharm. Biomed. Anal..

[26] eetmeter https://mijn.voedingscentrum.nl/nl/eetmeter.

[27] van Spronsen F.J., van Wegberg A.M., Ahring K. (2017). Key European guidelines for the diagnosis and management of patients with phenylketonuria. Lancet Diabetes Endocrinol..

[28] Mancilla V.J., Mann A.E., Zhang Y., Allen M.S. (2021). The adult phenylketonuria (PKU) gut microbiome. Microorganisms.

[29] Hooper L.V., Littman D.R., Macpherson A.J. (2012). Interactions between the microbiota and the immune system. Science.

[30] Cotillard A., Kennedy S.P., Kong L.C. (2013). Dietary intervention impact on gut microbial gene richness. Nature.

[31] Flint H.J., Scott K.P., Louis P., Duncan S.H. (2012). The role of the gut microbiota in nutrition and health. Nat Rev Gastroenterol Hepatol.

[32] Zhao Y., Tian G., Chen D. (2020). Dietary protein levels and amino acid supplementation patterns alter the composition and functions of colonic microbiota in pigs. Animal Nutrition.

[33] Radjabzadeh D., Boer C.G., Beth S.A. (2020). Diversity, compositional and functional differences between gut microbiota of children and adults. Sci. Rep..

[34] Amaretti A., Gozzoli C., Simone M. (2019). Profiling of protein degraders in cultures of human gut microbiota. Front. Microbiol..

[35] Pessione E. (2012). Lactic acid bacteria contribution to gut microbiota complexity: lights and shadows. Front. Cell. Infect. Microbiol..

