# Supplementary figures and images for: Differences in faecal microbiome composition between adult patients with UCD and PKU and healthy control subjects
Source: Mol Genet Metab Rep. 2021 Sep 8;29:100794. doi: 10.1016/j.ymgmr.2021.100794 (PMC8433284; doi:10.1016/j.ymgmr.2021.100794)

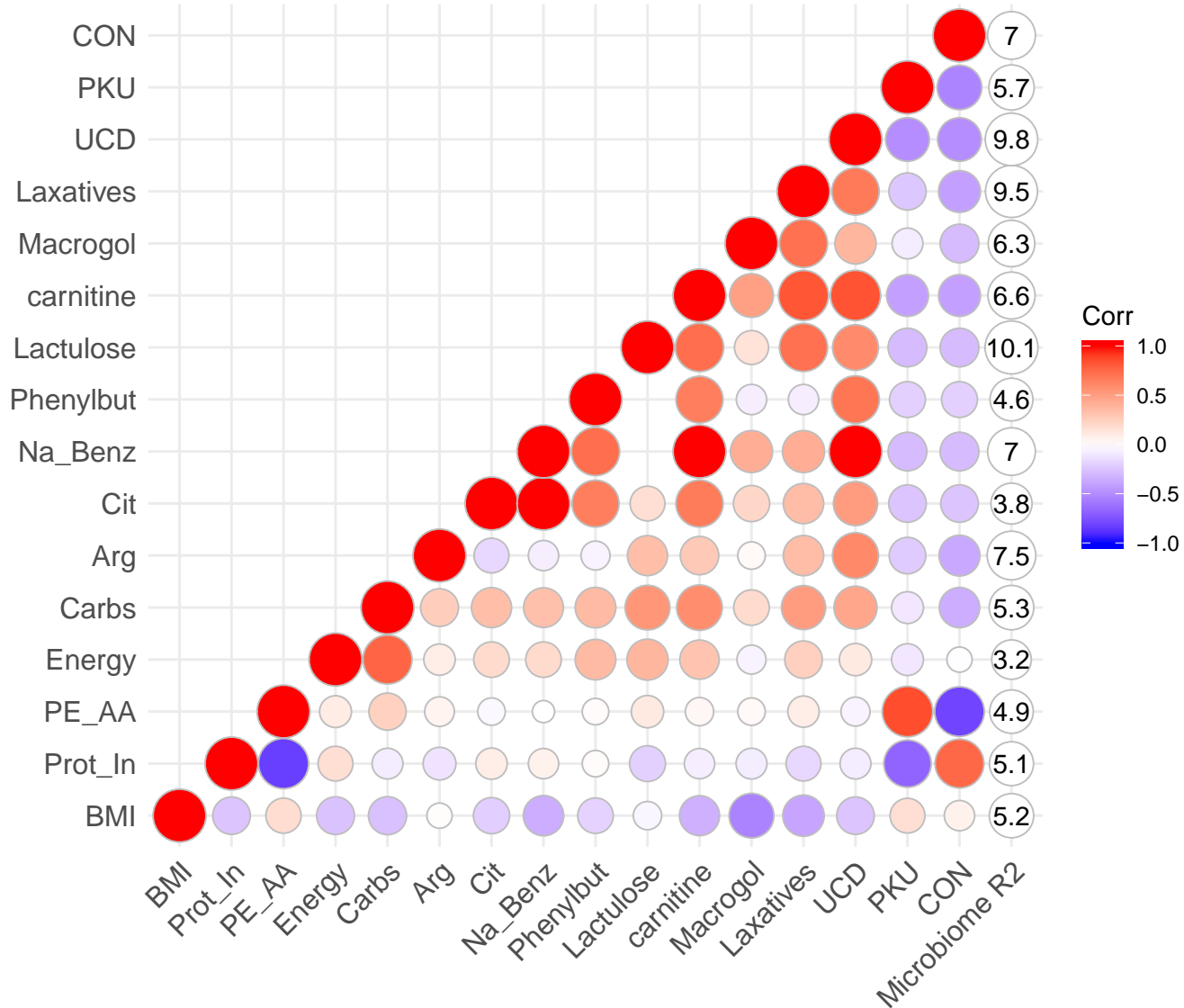

Supplement: The following are the supplementary data related to this article.Supplementary Fig. 4 — Visual correlation matrix: a graphical display of correlation, with confidence interval. Positive correlations are displayed in red and negative correlations in blue colour. Colour intensity and the size of the circle are proportional to the correlation coefficients. The most right column shows the explained variance (R2) in microbiome composition as determined by permutation MANOVA. [file mmc1.pdf]
