# Supplementary material for: Differences in faecal microbiome composition between adult patients with UCD and PKU and healthy control subjects
Source: Mol Genet Metab Rep. 2021 Sep 8;29:100794. doi: 10.1016/j.ymgmr.2021.100794 (PMC8433284; doi:10.1016/j.ymgmr.2021.100794)
Supplement: Supplementary file 1 — A layman explanation of microbiome terminology and data-analyzation. [file mmc4.docx]

**Supplementary file 1: A layman explanation of microbiome terminology and data-analyzation.**

**Abundance=** frequencies of operational taxonomic units (OTU’s).

**Relative abundance of species** refers to how common or rare a species is relative to other species in a defined location or community. Relative species abundance is calculated by dividing the number of species from one group by the total number of species from all groups.

**Alpha diversity** is the diversity of species within a single ecosystem or sample. This is used as a measure of biodiversity. The simplest measure is richness, the number of species (or OTUs) observed in the sample. Other metrics consider the abundances (frequencies) of the OTUs, for example to give lower weight to lower-abundance OTUs. Panels representing **alpha diversity measure** in our study are: Observed, Shannon and FPD. "How are the microbes balanced to each other?"

**Beta diversity=** variation of microbial communities between samples.

**Observed diversity**= total number of operational taxonomic units (OTU’s) observed; this is also known as **richness.**

**Operational taxonomic unit (OTU)=** a pragmatic definition to group individuals by similarity, or simply the group of organisms currently being studied.

**Shannon diversity index** = combines richness and diversity. It measures both the number of species and the inequality between species abundances. microbial index of diversity, a widely used index for comparing diversity between various habitats (Clarke and Warwick, 2001). The Shannon-Weiner index is based on measuring uncertainty (Barnes et al. 1998). This index takes into account two different aspects that contribute to the concept of diversity in a community: species richness and evenness.

**FPD=Faith’s Phylogenetic Diversity**, an alpha diversity metric accounting for genetic diversity. Phylogenetic diversity (“PD”) is a measure of biodiversity, based on phylogeny (the tree of life). Faith (1992) defined the phylogenetic diversity of a set of species as equal to the sum of the lengths of all those branches on the tree that span the members of the set. The branch lengths on the tree are informative because they count the relative number of new features arising along that part of the tree. This means that PD indicates “**feature diversity**” and “**option value**”. **Feature diversity** is the relative number of different features represented among species or other taxa. Biodiversity **option value** is the benefit biodiversity provides to us now in ensuring possible benefits for future generations.

**Species diversity:** tells us how evenly the microbes are distributed in a sample: "How are the microbes balanced to each other?"

**Species richness** is the number of different species in a sample: ‘How many microbes?’ (also see: Observed diversity).

**An example** of the difference between richness and diversity:

Two forests of each 100 trees consists of 3 species (oak, pine and elm). Forest 1 consists of 2 elms, 3 oaks, and 95 pine trees. Forest 2 consists of 25 Elms, 38 Oaks and 37 pine trees. Both Forests have the same richness (3 species), but forest 2 is more diverse (less chance to see lot of the same trees).

If you would plant a new forest (forest nr 3) with again 100 trees, and you will plant 20 elms, 28 Oaks, 30 pine trees and 22 birch trees, this forest will have a greater richness (more species) than forest 1 and 2. The diversity is also greater than forest 1, but comparable with the diversity of forest 2.
