# Supplementary material for: Differences in faecal microbiome composition between adult patients with UCD and PKU and healthy control subjects
Source: Mol Genet Metab Rep. 2021 Sep 8;29:100794. doi: 10.1016/j.ymgmr.2021.100794 (PMC8433284; doi:10.1016/j.ymgmr.2021.100794)
Supplement: Supplementary file 2 — HPLC Short Chain Fatty Acid Analysis. [file mmc5.docx]

**Supplementary file 2: HPLC Short chain fatty acid analysis**

### ***Materials***

External standard SCFA master mix (50 mM); containing, Formic acid, Acetic acid, Propionic acid and Butyric acid in Milli-Q water (Sigma Aldrich, Zwijndrecht, Netherlands). Internal Standard (IS), (Succinic acid 0.4 M) in Milli-Q, 12M Hydrochloric acid (37%), Diethyl Ether, 1M Sodium Hydroxide (NaOH) (Sigma Aldrich, Zwijndrecht, Netherlands). Running buffer: 20 mM NaH2 PO4 pH 2.2 (Sigma Alldrich, Zwijndrecht, The Netherlands), Acetonitrile (HPLC grade).

## *Methods*

*SCFA analysis*

Sample preparation and HPLC analysis of the fecal SCFA’s samples were carried out according to the method from De Beare *et al*. with some modifications.

*Standard and Sample preparation*

All standard points and the internal standard were prepared in milli-Q water. The 50 mM stock solution of the SCFA mix was aliquoted and stored at -20˚C until analysis. The external standard curve including the IS was prepared according to double dilute series ranging from 50 – 0 mM.

On a 200-300 mg (wet weight) stool sample a SCFA’s extraction procedure was performed. A second stool sample was used for the dry weight determination of the stool. In brief, 1 mL Milli-Q Water was added, together with 50µL IS, followed by a 15 min vortex step. A total of 100 µL HCl (12 M) was added to all samples and calibration preparations , followed by a 15s vortex step. Subsequently all samples were extracted twice with 7.5 mL di-ethylether for 20 minutes followed by an centrifugation step at 2000g for 5 minutes each. Both supernatants were pooled into a clean 30 mL tube and 500µL of 1M NaOH was added finally. After a short spin the aqueous phase was transferred to a new tube and 100 µL of HCl (12M) was added and vortexed.

*HPLC analysis*

A 300 µL sample was pipetted into a vial insert and 10 µL was injected for HPLC analysis.

All stool samples and 9 external calibration points were measured in one HPLC session run. Separation of the SCFA was carried out with a Hypersil Gold aQ column (150 mm × 4.6 mm, dp: 3 µm) using a Jasco quaternary pump (PU4285, Jasco Benelux, De Meern) at 30 **⁰**C. Variable flow conditions were applied for the phosphate/acetonitrile gradient (See Table 1). UV detection was done at 210 nm using a Jasco UV detector (UV4075, Jasco Benelux, De Meern). All samples were stored in a Jasco autosampler (AS4285, Jasco Benelux, De Meern) at 4 **⁰**C until analysis. For data interpretation Chrom-Nav chromatography software was used (Version 2.0, Jasco, de Meern).

The SCFA content was calculated by referring the SCFA/IS peak ratios to the ratios of the external standard curve. The final SCFA concentration was expressed in umol/g feces (wet weight). The dry weight was used for calculation of the factor wet/dry weight ratio by which the SCFA concentration was multiplied resulting in a dry stool SCFA concentration (umol/g).

Table 1

| Time (min) | A (%) | B (%) | Flow rate (mL/min) |
| --- | --- | --- | --- |
| 0.0 | 100 |  | 0.8 |
| 3.5 | 100 |  | 0.8 |
| 4.0 | 80 | 20 | 1.50 |
| 10.0 | 80 | 20 | 1.50 |
| 10.5 | 100 |  | 1.50 |
| 13.5 | 100 |  | 1.50 |
| 14.0 | 100 |  | 0.8 |
| 15.0 | 100 |  | 0.8 |

| Mobile phase:  A: 20 mm NaH_2_PO_4_  pH 2.2 |
| --- |
| B: acetonitrile 100%. |
